# Multilevel analysis of HIV related risk behaviors among heroin users in a low prevalence community

**DOI:** 10.1186/1471-2458-9-137

**Published:** 2009-05-12

**Authors:** Huizhen Li, William Goggins, Shui Shan Lee

**Affiliations:** 1 Stanley Ho Centre for Emerging Infectious Diseases, The Chinese University of Hong Kong, Hong Kong, PR China; 2 School of Public Health and Nethersole School of Nursing, The Chinese University of Hong Kong, Hong Kong, PR China

## Abstract

**Background:**

Injecting drug users (IDU) are at increased risk of human immunodeficiency virus (HIV) infection. Their HIV prevalence however varies from place to place and may not be directly linked with the level of individual risk. This study explores the relative importance of individual and community level characteristics in the practice of HIV-related risk behaviors in IDU in Hong Kong where the HIV prevalence has remained low at below 1%.

**Methods:**

Methadone clinics were used as the channel for accessing drug users in Hong Kong. HIV-related risk factors in drug users attending these clinics were retrieved from a questionnaire routinely administered to newly admitted and readmitted clients, and assessed using logistic regression and multilevel analyses.

**Results:**

Between 1999 and 2005, a total of 41196 person-admissions were recorded by 20 methadone clinics. Male gender, older age and new admissions in bigger clinics located in districts with older median age were more likely to have engaged in HIV related risk behaviors including heroin injection, needle sharing, unprotected sex and having multiple sex partners (p < 0.05).

**Conclusion:**

Multilevel analysis is a useful adjunct for determining the association between risk behaviors and both individual and community factors in IDUs, which can be demonstrated even in low HIV prevalence settings.

## Background

Injecting drug users (IDUs) are vulnerable to HIV infection both because of the sharing of contaminated injection equipment and their practice of high risk sexual behaviors. In Hong Kong, a metropolitan city in Southern China, only 5.6% of the 3400 HIV cases reported as of mid-2007 were IDUs, and their HIV prevalence has remained at no more than 1% in different settings.[1] However, some Chinese provinces have reported HIV prevalences of more than 20% in IDUs, and in most cities in the Pearl River Delta region adjacent to Hong Kong the estimated prevalences have ranged from 3–6%. [2-4] HIV outbreaks can potentially occur in future because some IDUs may have risky practices in China. A series of local studies conducted between 2000 and 2004 revealed that 25% of Hong Kong's IDUs had ever used drugs in Mainland China.[5] Other reports suggested that Mainland Chinese commercial sex workers (CSWs) who were also IDUs were less inclined to use condoms (50% vs 77%) and more likely to share needles (50% vs 20%), compared with non drug using CSWs.[4,6] Chances are that if IDUs in Hong Kong inject and share needles with Mainland Chinese IDUs in high HIV prevalence areas in China, or have unprotected sex with CSWs, HIV could easily spread. An assessment of the risk behaviors of IDUs in Hong Kong is important for predicting the potential of HIV spread, despite the low prevalence currently recorded locally.

IDUs constitute a marginalized and hard-to-reach population in society. They tend to socialize with their fellow IDUs rather than members of the general population. This unique social network configuration may shape their behavior patterns. Within the same group of IDUs, behaviors tend to be similar because of peer influence. The extent to which IDUs engage in risky behaviors (injection, needle sharing, syringe reuse, multiple sex partners and unprotected sex), and their individual demographic characteristics, such as age, gender, ethnicity, have been found to be correlated with HIV seropositivity. [7-9] A study conducted in the United States [10] considered IDUs with similar practices as members of a 'homogeneous cluster', and went on to describe the variation of HIV seroprevalence across clusters. Another study [11] reported that community characteristics derived from census data were predictors of HIV related risk among people residing in the same census tract. These multilevel studies were conducted in western countries, and so far no similar findings have been reported from Asian countries. In this study, we undertook to examine the variation of IDU associated risk behaviors at individual and community level in an Asian city, Hong Kong. Methadone clinics were used as the channel for accessing IDUs because of their high coverage of heroin users in the territory. The government-run clinics were set up in 1970s, and over the past three decades they have evolved to become focal points of neighborhoods for IDUs in different geographic areas. Service data available from these clinics were used to assess the relative importance of individual and community level characteristics on the practice of risk behaviors.

## Methods

This was a multi-site, institution based, cross-sectional study using data retrieved from results of a routine structured questionnaire administered on clients of the 20 methadone clinics in Hong Kong following informed consent. Institutional approval for access to the data was sought from the Department of Health, in compliance with the Personal Data (Privacy) Ordinance. The conduct of the study complied with the provisions of the Declaration of Helsinki. Contents of the questionnaire included anonymized personal data, and questions on drug taking habits, and sexual practices in the 12-month period prior to registration. There were two categories of registration in methadone clinics, new admission and readmission. New admissions referred to those clients who have never taken methadone treatment before, whereas readmissions were those who had enrolled in methadone treatment in the past, but had dropped out for over 28 days. Since each entry was anonymous, we could not match the readmissions with the new admissions of the same individuals. Therefore, in this study, only data from questionnaires on newly admitted drug users were used in logistic regression and multilevel analyses.

Risk behaviors were assessed to determine the vulnerability of drug users to HIV infection. Although early studies have determined the relative transmission efficiencies of HIV for each practice, [12-14] recent studies demonstrated the importance of sexual contact as a risk factor as compared to injection in IDUs.[15] Practice of injection, needle sharing, unprotected sex and multiple sex partners were assessed individually. Injection was defined as any report of injecting drug prior to registration, while needle sharing behavior was divided into current and past sharing practice. Unprotected sex referred to the use of condoms in less than 50% of the sexual activities in the preceding 12 months, while those reporting to have more than 1 sex partner in the same time period was defined as having multiple sex partners.

Two sets of variables were computed to determine their roles as predictors – at individual level and community level. Taking reference from variables that had been found to be significant in other studies, the following individual level predictors were used: gender, age and admission quarter/year. Age and its square were used to explore the non-linear association of age with HIV risk behaviors. Community level variables included population level data from each of the 18 administrative districts in Hong Kong obtained from the 2006 by-census. Only median age, median household income level, population density, and education level were available from this database. As income was highly correlated with education level, only income level was used in the analysis. In addition, the number of total admissions for each clinic was used as a community level variable reflecting the size of the IDU neighborhood.

Logistic regression was used to examine the effects of individual level predictor variables on the binary outcomes. Significance levels were reported for multivariate analyses and multilevel analyses with statistical significance set at 0.05. Age and age-squared were entered into the models as continuous variables and odds ratios were calculated for selected ages with age 20 as the reference. SPSS version 13.0 (SPSS Inc 2004) was used for the descriptive statistical analyses and to fit the logistic regression models. Multilevel logistic regression models were used to assess the independent predictive ability of both individual and community level covariates, and were fit using Supermix 1 statistical software (SSI Inc).

## Results

Additional file 1 provides a descriptive overview of the characteristics of our study population. From 1999 to 2005, there were totally 41196 person-admissions, of which 5184 were newly admitted drug users. The new admissions were predominantly male (77.6%) and aged below 30 (62.4%). They were younger than drug users on readmission (in which 53.7% were aged between 30 and 50). The yearly number of new admissions dropped steadily from around 1004 in 1999 to 482 in 2005, with a rebound to 965 in 2002.

The most frequently reported drug of abuse was heroin. More than 98% of the new admission clients reported the use of heroin alone or in combination with other drugs. Their commonest HIV related risk behaviors were injection and unprotected sex. While a majority of the new admissions preferred inhalation, smoking or sniffing, more than half of the readmission cases used injection as their major mode of heroin use. A quarter of the newly admitted heroin users reported current injection. Unclean syringe use was reported by 18% of new admissions, while the proportion among readmissions was 14%. Current needle sharing was reported in only 2% of the new admissions and readmissions, but some 7% of the readmissions reported past needle sharing. A slightly higher proportion of new admissions than readmissions reported having multiple sex partners while the reversed was true for unprotected sex in the preceding 12 months (10.1% vs 8.8%, *p *> 0.05 for multiple sex partners and 61.6% vs 63.7%, *p *= 0.02, odds ratio (OR): 0.91, 95% confidence interval (CI): 0.84–0.98 for unprotected sex).

Using multivariable logistic regression, 4 separate models – with injection, needle sharing, unprotected sex and multiple sex partners as outcomes – were used to assess HIV related risks. (Additional file 2) Male gender was strongly associated with heroin injection and having multiple sex partner (adjusted odds ratio (AOR): 1.27, 95%CI: 1.08–1.48, *p *= 0.004; AOR: 2.54, 95%CI: 1.91–3.38, *p *< 0.001 respectively) in all except the needle sharing model, in which only male gender was correlated with needle sharing behavior in univariate analysis. However, male drug users were less likely to have engaged in unprotected sex than female in the study population (AOR: 0.60, 95%CI: 0.52–0.68, *p *< 0.001). Age was also a significant predictor of heroin injection and unprotected sex. The positive coefficient for the age term and the negative coefficient for the age-squared term suggested a non-linear association, with more risky behaviors occurring in the middle of the age ranges (30 to 40 for injecting heroin use and 30 to 50 for unprotected sex). The time trend was evaluated by assessing the role of admission date. Later admission quarter/year was associated with more injection and lower tendency of having multiple sex partners (*p *< 0.001 in both models).

Preliminary analyses were performed on the data prior to multilevel modeling, the results of which confirmed the contextual structure of data and demonstrated the existence of between-community variation. In applying the multilevel model, age, gender, and admission were considered to exert fixed effects. Community-level variables (household income, clinic size, population density and median age of district population) were included as predictors of district-specific intercept, while no variables were used to predict the slope for time because no significant cross-level interactions were found in the preliminary analysis. Results of our models (Additional file 3) showed that individual variables (gender, age and admission time) remained as significant factors for predicting the risk behavior. Male gender was significantly associated with all four HIV risk behaviors, while the significance of age was seen only in the injection and unprotected sex models. Later admission was associated with slightly more injection practice and lower likelihood of multiple sex partners. After controlling for individual characteristics including age and gender, clients from bigger clinics, as defined by high caseload, were more likely to have injected heroin, shared needle and had sex without condom (*p *< 0.05). Clients from districts with older median age were more likely to have practiced risky sexual behaviors (*p *< 0.001).

## Discussion

This is the first study in an Asian population involving the analysis of data from methadone clinics to examine the variation of risk behaviors of drug users, using both logistic regression models and multilevel models. Results from our study demonstrated a variation of HIV risk behaviors across neighborhoods, as defined by the clinic locations where methadone users went for regular treatment. In Hong Kong, each methadone clinic functions like the center of a separate neighborhood. Clients of most clinics resided near the clinics at which they registered. Apparently, drug users spent most of their time around the clinic area and socialized with other drug users from the same neighborhood. It is not surprising, therefore, that they formed a local social network and were under the influence of their fellow drug users, leading to a similar pattern of drug taking behaviors. Associated with drug injection, needle sharing and unprotected sex, bigger clinic size was a predictive factor of risk behavior in our multilevel models. This result was consistent with studies elsewhere,[16,17] in which smaller social network implied lower levels of risky drug use behaviors. We did not, however, have relevant data for assessing the exact social network size of each drug user, and the inter-relationship between network size and with clinic size. The possible association of clinic size with HIV risk would need to be investigated in a study designed specifically for this purpose. As a harm reduction measure, the optimum size of methadone clinics is important, in order that maximum benefits of substitution treatment can be derived without engendering undesirable behavioral risks.

Some studies had examined the association between population characteristics and injection practice among IDUs.[11,18] It was shown in our study that an older median age in a district was a predictor of sexual risk. Median population age reflects the composition of population in the locale, as well as the education level and income level. People with older age were less likely to have been exposed to safer sex messages which had become standard publicity themes only after the beginning of the AIDS epidemic in early 80s. It may therefore be argued that condom use and multiple sex partners could in fact be more common in the older population, suggesting that drug users in such neighborhoods could be influenced to share the same practices. Furthermore, median population age could also be a surrogate for population density, since in Hong Kong the districts with older populations tend to be the more densely populated older parts of the city. In our study however population density was not found to be a significant predictor. Using multilevel analysis, we failed to find any association between population income and HIV risk. In our study, median household income was used as the surrogate, the level of which ranging between HKD9000 and HKD30000 (USD1 = HKD7.8). Since individual income level was not available and thus not controlled in the multilevel model, the analysis might not be robust enough to detect any effect of poverty on risk behaviors. Neighborhood variation in HIV prevalence has in fact been demonstrated in a previous study.[19] Among the neighborhood conditions, lower income was found to be associated with higher HIV prevalence among IDUs.[20] Overall, we were able to demonstrate a variation of HIV risk at community level. The generally low HIV rate in drug users in Hong Kong does not allow us to examine the correlation of such factors with HIV prevalence.

**Figure 1:**
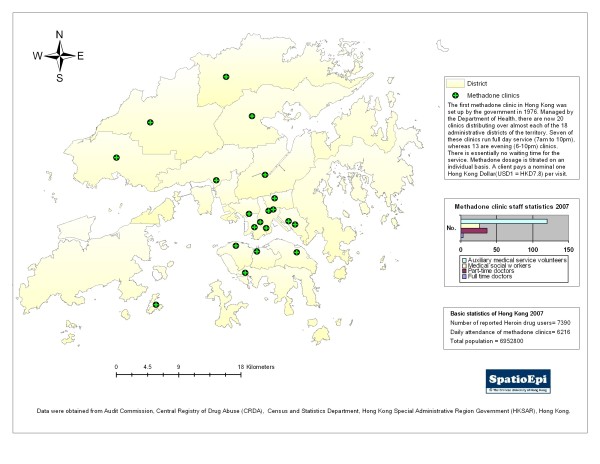
**Geographic distribution of methadone clinics in Hong Kong**.

On an individual level, our study showed a strong association of male gender with heroin injection, needle sharing and higher sexual risk, while age (30 to 40 for drug injection and older age for unprotected sex) also exhibited a positive association. Male gender and older age have been shown to be predictive factors of increased HIV risk in other studies.[21,22] A similar study using existing record to explore the behavioral change of IDUs in Hong Kong demonstrated a decline in needle sharing over the years,[23] an observation also made in our study. The low prevalence of needle sharing in our study population may be a result of the easy access to disposable syringes from local pharmacies. Also, the good coverage of methadone services has helped to reduce needle sharing behaviors.(Figure 1) Taken together, drug users' access to syringe and the provision of substitution treatment at the methadone services provided an explanation for the low HIV rate in Hong Kong.[24]

Our multilevel study of drug users carried certain limitations. First of all, Hong Kong is a small city with a total area of just 1000 Km^2 ^and with relatively 'homogenous' community characteristics. Unlike countries such as the United States in which the IDU population is often composed of a variety of ethnicities, almost all drug users in Hong Kong are Chinese. Variation of ethnicities has been reported to be related to HIV transmission in IDU,[25] a phenomenon that should be less important in Hong Kong. Our analysis illustrates community level variation in risk behaviors between clinics despite the 'homogenous' community characteristics and thus provides evidence that multilevel modeling could also be applied to community with less variability of the population profile. On the other hand, the study sample might not be representative of all drug users in the territory. Theoretically a representative sample could have been obtained by conducting street surveys in randomly selected sites in the city. [26-28] The feasibility of such sampling is low because IDUs constitute a hard-to-reach population, and social discrimination often creates an unfriendly environment for them to be accessed. Furthermore, their use of illicit drugs dissuades them to reveal their lifestyle to strangers.[29] Our solution to the sampling problem was to resort to existing service data.[30] In this connection, the government-run methadone treatment service in Hong Kong provides a particularly useful data source. The clinics have been in contact with an estimated 70% of all drug users.[31] Each methadone user is interviewed on admission and their demographic data, drug taking habits and sexual behaviors are recorded. With different opening hours, the 20 methadone clinics are widely distributed and are convenient for both jobless and employed clients. The major limitation is however the availability of small number of selected predefined fields for analyses. Another possible limitation is the self-reported nature of the data. Although it is mandatory for the questionnaire to be completed on admission, the self-reported behaviors may not be entirely reliable. As methadone users have to register their names and identity card numbers (anonymized in this study), there is the possibility that they are inclined to conceal their practices, though the prescription of methadone doses is not linked with reported behaviors. An underestimation of practice of risky behaviors is nevertheless a cause for concern. In some studies, however, self-reports of behaviors were found to be reliable in the collection of data on risk behaviors.[32,33]

## Conclusion

The increasing reports of HIV infections through injecting drug use among the HIV cases in Hong Kong raises the concern about the potential spread of the virus within this population and beyond.[34] Results from our study have provided an analytical description of the characteristics of the HIV related risk behaviors of IDUs. By elucidating the association between risk behaviors and both individual and community level factors, our approach may help predict the HIV risk of IDUs in a spatial context, which can in turn support effective allocation of resources to achieve prevention. We have demonstrated that the application of multilevel modeling could explain the contextual influences of individual and community factors on HIV related behavior in an Asian population, thereby calling for interventions to be geared towards this new angle in order to achieve effective prevention.

## Competing interests

The authors declare that they have no competing interests.

## Authors' contributions

HL conducted field study, collated the data, conducted all statistical analyses, and prepared the first draft of the manuscript. WG provided statistical support to the study. SSL conceptualized the study, coordinated all research activities and edited the manuscript. All authors have read the revised version of the manuscript and approved the final version.

## Pre-publication history

The pre-publication history for this paper can be accessed here:



## Supplementary Material

Additional File 1 **Characteristics of the study population**. The data provided shows the demographics, drug taking habits, and the practice of high risk behaviors in drug users in the study population. Click here for file

Additional File 2 **Predictors of HIV risk at individual level**. Results of logistic regression with OR (95%CI) for determining possible predictors of HIV risk. Click here for file

Additional File 3 **Predictors of HIV risk at two levels**. Results of multilevel analysis with OR (95%CI) (N = 5160) for determining possible predictors for HIV risk. Click here for file
