# Supplementary material for: Multilevel analysis of HIV related risk behaviors among heroin users in a low prevalence community
Source: BMC Public Health. 2009 May 12;9:137. doi: 10.1186/1471-2458-9-137 (PMC2687448; doi:10.1186/1471-2458-9-137)
Supplement: Additional File 1 — Characteristics of the study population. The data provided shows the demographics, drug taking habits, and the practice of high risk behaviors in drug users in the study population. [file 1471-2458-9-137-S1.doc]

| Characteristics | | | New admission | | Readmission | |
| --- | --- | --- | --- | --- | --- | --- |
| Demographics | | |  |  |  |  |
|  | Gender | | N=5185 | % | N=36011 | % |
|  |  | Male | 4025 | 77.6 | 31371 | 87.1 |
|  |  | Female | 1160 | 22.4 | 4640 | 12.9 |
|  | Age | |  |  |  |  |
|  |  | 20 or below | 793 | 15.3 | 660 | 1.8 |
|  |  | 21 to 30 | 2441 | 47.1 | 8892 | 24.7 |
|  |  | 31 to 40 | 1297 | 25.0 | 9400 | 26.1 |
|  |  | 41 to 50 | 369 | 7.1 | 9953 | 27.6 |
|  |  | 51 to 60 | 138 | 2.7 | 5054 | 14.0 |
|  |  | Above 60 | 147 | 2.8 | 2052 | 5.7 |
| Drug taking Profile | | |  |  |  |  |
|  | Heroin only | | 5084 | 98.1 | 35088 | 97.4 |
|  | Heroin and Opiate/Opium | | 1 | 0.0 | 4 | 0.0 |
|  | Heroin and others | | 21 | 0.4 | 493 | 1.4 |
|  | Opiate/Opium only | | 57 | 1.1 | 105 | 0.3 |
|  | Others only | | 22 | 0.4 | 321 | 0.9 |
| Major method of Heroin use | | | N=5106 | % | N=35585 | % |
|  | I.M./I.V.^a^ | | 1323 | 25.9 | 18705 | 52.6 |
|  | Inhalation/smoking/sniffing | | 3781 | 74.1 | 16864 | 47.4 |
|  | Oral | | 2 | 0.0 | 16 | 0.0 |
| HIV risk behavior (Heroin users) | | | N=5106 | % | N=35585 | % |
|  | Injection history | |  |  |  |  |
|  | Current injection | | 1323 | 25.9 | 18705 | 52.6 |
|  |  | | N=1323 | % | N=18705 | % |
|  | Current unclean syringe use | | 233 | 17.6 | 2544 | 13.6 |
|  | Past needle sharing | | 68 | 5.1 | 1300 | 7.0 |
|  | Current needle sharing | | 28 | 2.1 | 386 | 2.1 |
|  | | | Sex behavior | % | N=36011 | % |
|  | Multiple sex partners | | 525 | 10.1 | 3154 | 8.8 |
|  |  | | N=3115 | % | N=18047 | % |
|  | Unprotected sex | | 1919 | 61.6 | 11492 | 63.7 |
| New admission: first time registering clients; Readmission: clients who have dropped out of the methadone service for 28 days or more | | | | | | |
| ^a^I.M.: intramuscular injection; I.V.: intravenous injection | | | | | | |
