# Supplementary material for: Multilevel analysis of HIV related risk behaviors among heroin users in a low prevalence community
Source: BMC Public Health. 2009 May 12;9:137. doi: 10.1186/1471-2458-9-137 (PMC2687448; doi:10.1186/1471-2458-9-137)
Supplement: Additional File 2 — Predictors of HIV risk at individual level. Results of logistic regression with OR (95%CI) for determining possible predictors of HIV risk. [file 1471-2458-9-137-S2.doc]

| Predictors of risk | | Injection model | | | | Needle sharing model | | | | Unprotected sex model | | | | | Multiple sex partner model | | |
| --- | --- | --- | --- | --- | --- | --- | --- | --- | --- | --- | --- | --- | --- | --- | --- | --- | --- |
|  |  | Odds Ratio | 95% CI | *p* value | | Odds Ratio | | 95% CI | *p* value | Odds Ratio | | 95% CI | *p* value | | Odds Ratio | 95% CI | *p* value |
| Male | | 1.19 | 1.02-1.38 | | *p*=0.03^**^ | | 1.72 | 0.97-3.04 | *p*=0.062 | 0.64 | | 0.56-0.74 | | *p*<0.001^***^ | 2.57 | 1.93-3.401 | *p*<0.001^***^ |
| Age^ | |  |  | | *p*<0.001^***^ | |  |  | *p*=0.21/0.14 |  | |  | | *p*<0.001^***^ |  |  | *p*=0.11/0.86 |
|  | 20 | Referent | |  | |  | |  |  | Referent | | | |  |  |  |  |
|  | 30 | 2.29 | 2.17-2.43 | |  | |  |  |  | 1.77 | 1.73-1.80 | | |  |  |  |  |
|  | 40 | 2.36 | 1.89-2.96 | |  | |  |  |  | 2.10 | 1.94-2.26 | | |  |  |  |  |
|  | 50 | 1.09 | 0.66-1.82 | |  | |  |  |  | 1.67 | 1.40-1.98 | | |  |  |  |  |
|  | 60 | 0.23 | 0.09-0.56 | |  | |  |  |  | 0.89 | 0.65-1.21 | | |  |  |  |  |
| Admission quarter/year | | 1.04 | 1.03-1.05 | | *p*<0.001^***^ | | N/A | N/A | *p*=0.84 | 1.01 | 1.01-1.02 | | | *p*<0.001 | 0.97 | 0.96-0.98 | *p*<0.001^***^ |
| N/A: Not Applicable; ^: Age square was introduced into the analysis in order to explore the non-linear correlation of age and risk. Both variables (age and age square) were continuous, and OR was calculated for specific age. **: *p*<0.01 in multivariate logistic regression; ***: *p*<0.001 in multivariate logistic regression. | | | | | | | | | | | | | | | | | |
