# Supplementary material for: Multilevel analysis of HIV related risk behaviors among heroin users in a low prevalence community
Source: BMC Public Health. 2009 May 12;9:137. doi: 10.1186/1471-2458-9-137 (PMC2687448; doi:10.1186/1471-2458-9-137)
Supplement: Additional File 3 — Predictors of HIV risk at two levels. Results of multilevel analysis with OR (95%CI) (N = 5160) for determining possible predictors for HIV risk. [file 1471-2458-9-137-S3.doc]

| Predictors of risk | | Injection model | | | Needle sharing model | | | Unprotected sex model | | | Multiple sex partner model | | |
| --- | --- | --- | --- | --- | --- | --- | --- | --- | --- | --- | --- | --- | --- |
|  |  | Odds ratio | 95% CI | *p* value | Odds ratio | 95% CI | *p* value | Odds ratio | 95% CI | *p* value | Odds ratio | 95% CI | *p* value |
| Male | | 1.27 | 1.08-1.49 | *p*=0.003 | 1.84 | 1.04-3.27 | *p*=0.04 | 0.60 | 0.52-0.69 | *p*<0.001 | 2.98 | 2.24-3.97 | *p*<0.001 |
| Age^ | |  |  | *p*<0.001 |  |  | *p*=0.27/0.16 |  |  | *p*<0.001 |  |  | *p*=0.45/0.36 |
|  | 20 | Referent | |  |  |  |  | Referent | |  |  |  |  |
|  | 30 | 1.95 | 1.83-2.07 |  |  |  |  | 2.03 | 1.99-2.07 |  |  |  |  |
|  | 40 | 1.81 | 1.43-2.29 |  |  |  |  | 2.89 | 2.65-2.15 |  |  |  |  |
|  | 50 | 0.80 | 0.47-1.37 |  |  |  |  | 2.86 | 2.36-2.47 |  |  |  |  |
|  | 60 | 0.17 | 0.07-0.44 |  |  |  |  | 1.97 | 1.40-2.78 |  |  |  |  |
| Admission quarter/year | | 1.03 | 1.02-1.04 | *p*<0.001 | 0.99 | 0.97-1.02 | *p*=0.62 | 1.00 | 0.99-1.01 | *p*=0.96 | 0.98 | 0.97-1 | *p*=0.009 |
| Median household income | | 0.99 | 0.88-1.12 | *p*=0.90 | 0.92 | 0.63-1.36 | *p*=0.70 | 1.03 | 0.92-1.14 | *p*=0.64 | 0.98 | 0.84-1.16 | *p*=0.86 |
| Clinic size | | 1.05 | 1.01-1.09 | *p*=0.02 | 1.23 | 1.08-1.41 | *p*=0.002 | 1.04 | 1.00-1.08 | *p*=0.04 | 0.98 | 0.92-1.05 | *p*=0.59 |
| Median age | | 1.00 | 0.98-1.03 | *p*=0.86 | 1.05 | 0.98-1.12 | *p*=0.18 | 1.06 | 1.03-1.07 | *p*<0.001 | 1.08 | 1.05-1.12 | *p*<0.001 |
| Population density | | 1.00 |  | *p*=0.18 | 1 | 0.99-1.00 | *p*=0.43 | 1.00 |  | *p*=0.22 | 1 |  | *p*=0.77 |
| ^: Age square was introduced into the analysis in conjunction with age in order to explore the non-linear correlation of age and risk. Both variables were continuous, and only OR for specific age was calculated. Clinic size, median age, household income and population density are continuous variables, while the unit for median household income was 10,000 Hong Kong dollars, clinic size in thousand people and population density in thousand people/Km^2^. | | | | | | | | | | | | | |
